# Refining the content and design of an alcohol reduction app, *Drink Less*, to improve its usability and effectiveness: a mixed methods approach

**DOI:** 10.12688/f1000research.51416.2

**Published:** 2021-11-25

**Authors:** Claire Garnett, Olga Perski, Susan Michie, Robert West, Matt Field, Eileen Kaner, Marcus R. Munafò, Felix Greaves, Matthew Hickman, Robyn Burton, Jamie Brown

**Affiliations:** 1 Department of Behavioural Science and Health, University College London, London, WC1E 7HB, UK; 2 Department of Clinical, Educational and Health Psychology, University College London, London, WC1E 7HB, UK; 3 Department of Psychology, University of Sheffield, Sheffield, S1 2LT, UK; 4 Population Health Sciences Institute, Newcastle University, Newcastle upon Tyne, NE2 4AX, UK; 5 School of Psychological Science, University of Bristol, Bristol, BS8 1TU, UK; 6 Department of Primary Care and Public Health, Imperial College London, London, W6 8RP, UK; 7 Public Health England, London, SE1 8UG, UK; 8 Population Health Sciences, Bristol Medical School, University of Bristol, Bristol, BS8 1UD, UK

**Keywords:** smartphone app, development, refinement, alcohol, digital, behaviour change, Drink Less app

## Abstract

**Background:** Digital interventions have the potential to reduce alcohol consumption, although evidence on the effectiveness of apps is lacking. *Drink Less* is a popular, evidence-informed app with good usability, putting it in a strong position to be improved upon prior to conducting a confirmatory evaluation. This paper describes the process of refining *Drink Less* to improve its usability and likely effectiveness.

**Methods:** The refinement consisted of three phases and involved qualitative and quantitative (mixed) methods: i) identifying changes to app content, based on findings from an initial evaluation of *Drink Less*, an updated review of digital alcohol interventions and a content analysis of user feedback; ii) designing new app modules with public input and a consultation with app developers and researchers; and iii) improving the app’s usability through user testing.

**Results:** As a result of the updated review of digital alcohol interventions and user feedback analysis in Phase 1, three new modules: ‘Behaviour Substitution’, ‘Information about Antecedents’ and ‘Insights’, were added to the app. One existing module – ‘Identity Change’ – was removed based on the initial evaluation of *Drink Less*. Phases 2 and 3 resulted in changes to existing features, such as improving the navigational structure and onboarding process, and clarifying how to edit drinks and goals.

**Conclusions:** A mixed methods approach was used to refine the content and design of *Drink Less*, providing insights into how to improve its usability and likely effectiveness. *Drink Less* is now ready for a confirmatory evaluation.

## Introduction

Digital interventions have much promise in supporting health-related behaviour change; those for alcohol have been found to be effective at reducing hazardous and harmful alcohol consumption ( Kaner *et al*., 2017; Riper *et al*., 2018). Digital interventions include websites, wearable devices, and smartphone apps. Apps have the advantage of being available to deliver support when and where needed, with smartphones being the most important device used to access the internet in the United Kingdom (UK) in 2020 ( Ofcom; Critical Research, 2020). However, the majority of the alcohol-related apps available have been developed without reference to scientific evidence or theory ( Crane *et al*., 2015), and evidence on the effectiveness of apps to reduce alcohol consumption is lacking. A recent Cochrane evidence review ( Kaner *et al*., 2017) found that digital interventions may reduce alcohol consumption when compared with a control (i.e. screening, assessment, printed or onscreen health or alcohol-related information). However, only one of the 42 randomised controlled trials (RCTs) evaluated a smartphone app, and this study was conducted among university students in Sweden, not in a general population sample ( Gajecki *et al*., 2014). Therefore, despite the availability of hundreds of alcohol-related apps in commercial app stores, none so far have been evaluated in an RCT among the general population of adult drinkers in the UK. The lack of current evidence on the effectiveness of alcohol reduction apps highlights the need for a robust evaluation strategy of an evidence- and theory-informed alcohol reduction app.

Digital interventions tend to have multiple components, which pose a number of issues relating to initial evaluation with a RCT. These issues include understanding which components are driving intervention effectiveness, building in the ability to refine the intervention over time, making timely decisions about what components to retain in the intervention, and updating the intervention to keep pace with the rate of change of software. The Multiphase Optimisation Strategy (MOST) is an experimental approach for efficiently and systematically developing and optimising multi-component interventions within pre-specified constraints, such as financial ( Collins *et al*., 2005, 2007, 2011, 2014). MOST can be simplified into a three-step process of: screening (efficient identification of intervention components for inclusion in the ‘first draft’ of the intervention); refining (fine-tuning of the selected components in the ‘first draft’); and confirming (evaluating the ‘final draft’ of the intervention in a standard RCT) ( Collins *et al*., 2007). The initial screening and refining steps allow researchers to understand the effects of the individual intervention components and create an optimal intervention. MOST recognises that RCTs are the gold standard for assessing the effectiveness of an intervention as a package in its ‘final draft’, though highlights the importance of this being the last step after the intervention has been refined through experimentation. By following MOST, researchers are likely to avoid the (typically very slow) cycle of intervention – RCT – post hoc analyses – revision of intervention – RCT.

*Drink Less* is a freely available, evidence- and theory-informed app that aims to help the general population of people who are hazardous or harmful drinkers to reduce their alcohol consumption ( “Drink Less on iTunes store,” n.d.; Garnett *et al*., 2019a). *Drink Less* was guided by MOST ( Collins *et al*., 2005, 2007) and the first screening step was conducted between 2014 and 2016. The screening involved a systematic and transparent development process ( Garnett *et al*., 2019a) involving users across a range of socioeconomic groups ( Crane *et al*., 2017), and a randomised factorial screening trial ( Crane *et al*., 2018). The aim of the screening trial was to identify the individual components, or combinations of components, within the multi-component intervention that affect change and to screen out the ineffective ones ( Collins *et al*., 2014). The screening trial found no clear evidence for simple main effects but did find evidence that two-way combinations of certain components resulted in greater reductions in alcohol consumption, and there was an overall decline in weekly alcohol consumption among all trial users of 3.8 standard UK units ( Crane *et al*., 2018). Furthermore, *Drink Less* appears to have favourable usability amongst its users: the app was launched in May 2016 and has since been downloaded by over 62,000 unique users and has an average 4.3/5 star rating (as of January 2021) in the Apple App Store.

Since *Drink Less* is a popular, evidence-informed app ( Crane *et al*., 2018; Garnett *et al*., 2019a), a confirmatory evaluation is warranted. To ensure the evaluation was of optimal content and design, it was refined in line with the second step of MOST. This ‘refining’ step was important to fine-tune the selected components from the first version and ensure the app was in an optimal state prior to conducting a resource-intensive confirmatory evaluation. This refinement process is typically based on experimentation of the intervention ( Collins *et al*., 2011); however, other study designs may also be used. Here, we describe the three-phase, mixed methods process of refining the *Drink Less* app to improve its usability and likely effectiveness across socioeconomic groups, in preparation for its evaluation in an RCT.

## Methods

### Ethics statement

All procedures performed in studies involving human participants were in accordance with the ethical standards of the institutional research committee and with the 1964 Helsinki declaration and its later amendments or comparable ethical standards. Ethics approval was granted by the University College London’s Research Ethics Committee under the “The optimisation and implementation of interventions to change behaviours related to health and quality of life” project (CEHP/2016/556).

### Refinement process

**Figure 1.**
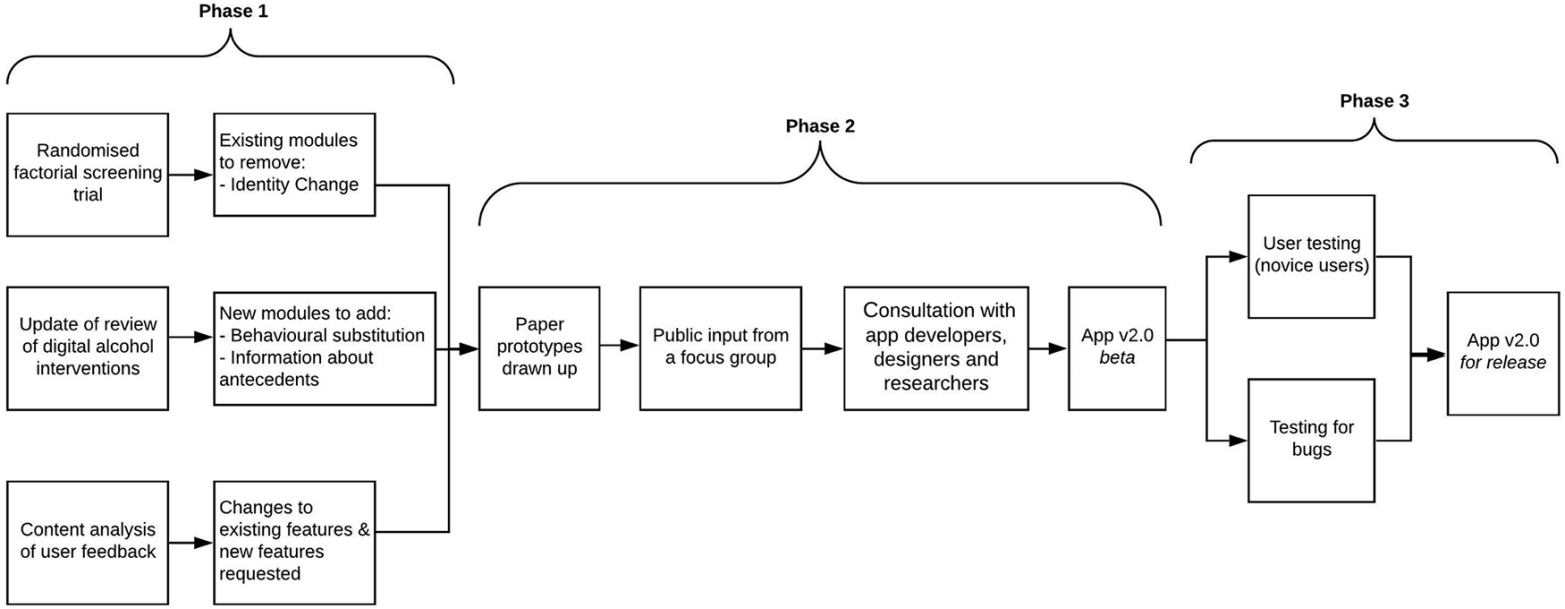
Process of refining the *Drink Less* app.

The refinement of the app consisted of three phases and involved a combination of qualitative and quantitative (mixed) methods: (i) identifying changes to the content of the app, (ii) design and translation of new content into app modules, and (iii) improving the usability of the app. The refinement began in January 2018 and the project ended in July 2019. The final version of the app (v2) was launched in January 2020. Figure 1 shows an overview of the process of refining the *Drink Less* app.

The development of the *Drink Less* app used the COM-B model of behaviour, which posits that behaviour results from interactions between ‘capability’, ‘opportunity’ and ‘motivation’, to provide an overarching theoretical structure ( Michie *et al*., 2014). The original version of *Drink Less* consisted of six modules: (i) Goal Setting; (ii) Self-Monitoring and Feedback; (iii) Normative Feedback; (iv) Cognitive Bias Retraining; (v) Action Planning; and (vi) Identity Change (details of these modules are reported in full elsewhere ( Garnett *et al*., 2019a)).

User input was included in every phase of the refinement process, which is important for digital interventions as they commonly have low rates of engagement and high rates of attrition ( Eysenbach, 2005; Helander *et al*., 2014). User input helps to ensure that a digital intervention has engaging content, and good acceptability and usability. We followed the principles of user-centred design, a systematic method of app development that focuses on the relevance, needs, and preferences of the user at every stage of the development process ( Mccurdie *et al*., 2012).

### Phase 1: Changes to the content of the app

Priority changes to the content of the app were identified based on findings from the randomised factorial screening trial of *Drink Less* ( Crane *et al*., 2018), an update of a review of digital alcohol interventions ( Kaner *et al*., 2017), and a content analysis of user feedback.

*Findings from the randomised factorial screening trial*

The randomised factorial screening trial of *Drink Less* with 672 excessive drinkers found that certain combinations of intervention modules produced modest improvements in alcohol-related outcomes after four weeks, though no clear evidence for main effects (published in full elsewhere ( Crane *et al*., 2018)). Bayes factors are a measure of strength of evidence and can distinguish between two interpretations of a non-significant result: i) support for the null hypothesis of ‘no effect’ and ii) data are insensitive to detect an effect ( Dienes, 2014; Jeffreys, 1961). Bayes factors also allow researchers to ‘top-up’ their results from one trial with additional data collected ( Rouder, 2014). In the factorial trial, Bayes factors showed the data to be insensitive to detect a main effect (d = 0.22) of individual modules so an update was conducted to analyse additional trial outcome data collected through extended recruitment. This update is published in full elsewhere ( Garnett *et al*., 2019b). After the required sample size was reached (n = 672), additional data from new participants (n = 1914) were collected over five months (available in *Underlying data:* https://osf.io/nad4e) Bayes factors with a half-normal distribution and priors set to the point estimates from the initial 672 participants were calculated.

*Update of review of digital alcohol interventions*

A Cochrane evidence review on the effectiveness of digital alcohol interventions found that ‘Behaviour substitution’ (prompt substitution of drinking with a neutral behaviour) was associated with intervention effectiveness ( Garnett *et al*., 2018b; Kaner *et al*., 2017) and worthy of inclusion in the next version of *Drink Less.* However, as the field of research on digital interventions is expanding rapidly, an update of the meta-regression on the behaviour change techniques (BCTs) associated with intervention effectiveness in the Cochrane systematic review on the personalised digital interventions for reducing excessive alcohol consumption was conducted. The same search strategy, inclusion criteria, and procedure as in the published Cochrane evidence review ( Kaner *et al*., 2017) was used with the search strategy run on 7 ^th^ March 2018 from the date of the last search (March 2017). Studies were RCTs with the outcome measure of quantity of alcohol consumption and participants were community-dwelling individuals who were identified as hazardous or harmful drinkers. The intervention had to target alcohol consumption of related problems in the drinker and be delivered through a digital device. A total of 12 new studies met the inclusion criteria, and were added to the 42 trials from the published Cochrane review, and were coded for BCTs using an established and reliable method – the BCT Taxonomy v1 with 93 distinct BCTs ( Michie *et al*., 2013). Meta-regressions were conducted to assess the association of the BCTs with intervention effectiveness. Effect sizes were based on a random effects model where each unstandardized effect was the mean difference between the digital intervention and control (expressed in grams of alcohol consumed per week, at the longest follow-up time point). Independent associations were investigated with an adjusted meta-regression analysis where all of the BCTs that had a meaningful association (defined as a regression coefficient, B>23 ( Kaner *et al*., 2007)) with intervention effectiveness in the unadjusted models were included. The BCTs of ‘Information about antecedents’ (B = −58.09, 95% CI = −91.29, −24.88) and ‘Credible source’ (B = −32.82, 95% CI = −58.15, −7.49) were independently associated with the effectiveness of digital interventions to reduce alcohol consumption.

The ‘Credible source’ (present communication from a credible source in favour of or against the behaviour) was already present in *Drink Less.* Therefore, this update concluded that ‘Information about Antecedents’ (which provides information about social and environmental situations and events, emotions, and cognitions that reliably predict drinking) should be added to the refined version of *Drink Less.* A short report on the update is available as *Extended data* ( https://osf.io/azpc2/) .

*Content analysis of user feedback*

User feedback typically contains positive and negative feedback on content, functionality, and quality of an app ( Khalid *et al*., 2015; Vasa *et al*., 2012). This feedback can help to identify key areas for change to improve the app’s usability and acceptability and provide a rich source of information to support app development from a user perspective ( Mudambi and Schuff, 2010).

User feedback on *Drink Less* from 18 ^th^ May 2016 (when the app was launched) until 28 ^th^ November 2018 (when the latest analysis of user feedback was conducted) was anonymised and collated (n = 480). The feedback was received via the Apple App Store user reviews (n = 116) and via email to the *Drink Less* support email address (n = 364). Content analysis was used to identify and report the key themes within the data ( Braun and Clarke, 2006), as well as to quantify the data in terms of the frequency of different themes ( Vaismoradi *et al*., 2013). The initial set of categories was selected according to categories used to classify users’ reviews for software maintenance and evolution ( Panichella *et al*., 2015). These are: 1) Information seeking (sentences related to attempts to obtain information or help from other users or from developers); 2) Information giving (sentences that inform or update users or developers about an aspect related to the app); 3) Feature request (sentences expressing ideas, suggestions or needs for improving or enhancing the app or its functionalities); and 4) Problem discovery (sentences describing issues with the app). Sub-themes within these categories were then generated inductively from the data, and the user feedback was coded against these initial sub-themes in an iterative process. Each case of user feedback could be coded multiple times. The most common category was ‘Information giving’ (35.1%, with the majority providing overall positive feedback on the app), followed by ‘Feature request’ (32.0%), then ‘Problem discovery’ (14.5%), ‘Information seeking’ (14.1%) and ‘Other’ (4.4%). The most frequent requests for changes to existing features were “changes to add drinks feature”, “ability to update normative feedback data”, “calendar to start on Monday”. Common new features requested were having an android version, international availability and a running total for units. The most common sub-themes in ‘Problem discovery’ were “Clocks changing” and “Bug with drinks data when time zone changes”. In the ‘Information seeking’ category, how to navigate to the mood diary and edit existing drinks entries were the most common sub-themes. A number of the sub-themes in this category were related to clarification on how to edit entries (e.g. mood diary, ABV, cost, volume) and navigation (e.g. drinking calendar). The content analysis of user feedback identified a list of high priority changes (e.g. changes to add drinks feature; ability to update normative feedback data; calendar to start on Monday; fix bugs with drinks data when time zone changes) that should be made to the existing app modules to improve the design and functionality of the app to refine it in terms of acceptability and usability. The full report of the user feedback analysis is available in *Extended data* ( https://osf.io/vdmsk/).

### Phase 2: Design and translation of new content into app modules

Phase 2 involved public input on paper prototypes (rough drawings of the user interface). This was considered alongside the feasibility of development and through consultation with app developers, designers and digital behaviour change researchers.

*Design principles*

As with the original development of *Drink Less* ( Garnett *et al*., 2019a), a number of design principles were followed to translate the new content of the app into modules that were coherent and appealing to use ( Garnett *et al*., 2019a). This included: a visually appealing and professionally designed app ( Garnett *et al*., 2015; Perski *et al*., 2016; Ubhi *et al*., 2015); easy to use, intuitive and consistent navigation ( Ubhi *et al*., 2015); credibility of the information presented ( Perski *et al*., 2016; Ubhi *et al*., 2015); and minimal text, free of scientific jargon ( Ubhi *et al*., 2015).

*Paper prototypes*

New app content was first proposed as a series of paper wireframe prototypes with multiple design options (available as *Extended data:* https://osf.io/mu7sw/) created by two members of the research team (CG and OP). The creation of paper prototypes for new modules was informed by how the new content had been implemented in other digital interventions ( Garnett *et al*., 2018b) and health-related apps. The paper prototypes differed in their design and functionality, whilst remaining consistent with the design principles.

*Public involvement through an Alcohol Discussion Group*

A meeting was held in November 2018 with the UK Centre for Tobacco and Alcohol Studies (UKCTAS) Alcohol Discussion Group, a public involvement group comprising approximately 20 people who consumed alcohol and lived locally to Stirling, Scotland. Two researchers (CG and OP) facilitated the meeting; neither had established a relationship with the public involvement group prior to the meeting. CG has a MSc in Research Methods in Psychology and has previously facilitated a workshop, and OP is a mixed-methods researcher and has conducted several interviews, focus groups and workshops as part of her doctoral and post-doctoral work. The meeting lasted two hours and the aim was to get input, comments and advice from this stakeholder group on various research materials, such as the content of the app and the paper prototypes for the new modules. A brief overview of the app and the research conducted to date was presented and then a group discussion was had about the new app features and how they could be designed in the app, along with general feedback on the app and upcoming research priorities. Notes were taken by CG and OP to summarise the group discussion with no direct quotes taken. Verbal permission was given by the UKCTAS Alcohol Discussion Group to take an audio recording of the session solely for the purpose of informing the summary notes (these summary notes are provided in *Underlying data*: https://osf.io/9ca37/).

*Consultation with app designers, developers and researchers*

App designers, developers and researchers with expertise in digital behaviour change interventions who were known to the research team were emailed and invited to take part in an online consultation on the quality and feasibility of the paper prototypes (e.g., the relative cost of different options). The aim was to identify the most favourable option for the more complicated changes arising from Phase 1. In Round 1, a total of 13 app designers, developers and researchers completed an online survey and nine completed the survey in Round 2. The survey instruments for each survey round are provided in *Extended data* (Round 1: https://osf.io/w3tx8/ , Round 2: https://osf.io/4xa73/) and the results for each survey are provided in *Underlying data* (Round 1: https://osf.io/jxvns/, Round 2: https://osf.io/vt3ub/). Written informed consent was obtained for participation and publication of anonymised data.

The final decisions were based on the preference of the majority and discussions with the app developer to assess ease of development, including cost. This informed the final app briefs provided to the developer for the refined version of the app, ready for user testing in Phase 3.

### Phase 3: Improving the usability of the app

Phase 3 involved user testing of the refined version of *Drink Less* and further internal bug testing.

*User testing of the resulting refined version from Phase 1 and 2*

The aim was to refine the usability and acceptability of the updated *Drink Less* app to inform changes to *Drink Less.* A think-aloud study was conducted using a person-based approach to elicit and observe user reactions among novice users of the updated app (version 2.0), and paper prototype designs for the ‘Insights’ module ( Yardley *et al*., 2015). A semi-structured interview was conducted after the think-aloud procedure with questions on: (i) participants’ expectations of the app, (ii) desired improvements or changes, and (iii) acceptability of the app ( Sekhon *et al*., 2017). The briefing interview guide, ‘think aloud interview’ guide, and semi-structured interview guide are provided in a brief report of the study (see *Extended data*: https://osf.io/wzvt8/).

Participants were recruited from London, Bristol, Sheffield and Newcastle using ‘ Call for Participants’, social media and an online classified and social community website and approached via email. Participants had to be aged 18 and over, have an Alcohol Use Disorder Identification Test (AUDIT) score of 8 or more (indicative of excessive drinking ( Babor *et al*., 2001)), and be interested in using an app to support health-related behaviour change. Purposive sampling was used to ensure participants of low socioeconomic status (SES) were adequately represented. This is critical to ensure that digital interventions at the very least do not exacerbate health inequalities, and hopefully reduce them. Including people from low SES groups in the development of the digital smoking cessation intervention, StopAdvisor, resulted in the intervention being more effective for those from low (in comparison with those from high) SES groups ( Brown *et al*., 2012). This highlights the importance of having input from users from low SES groups to maximise the acceptability and usability of the app across the social spectrum.

There were 18 participants that took part, which met the threshold recommended for a usability test to detect most of the usability problems ( Nielsen, 1994). Half of participants (n = 9) were either: unemployed; employed in a routine or manual occupation; or had no post-16 educational qualifications. No-one refused to participate or dropped out. The mean age of the whole sample was 33 years old (range: 19 to 65) and 44% were female (n = 8). The mean interview length was 52 minutes (range: 28 to 81). Data were collected in private rooms within university buildings (University College London, Bristol University, University of Sheffield, and Newcastle University) with no-one else present besides the participant and researcher. Data were collected between 4 ^th^ April and 22 ^nd^ May 2019.

Participants were briefed on the purpose of the study and understood that it was a research project for CG. No relationship with the participants was established prior to study commencement. Ethical approval had been granted, participants reviewed the participant information sheet prior to giving their written informed consent for participation in the study, and for their anonymised data to be used in future research. Participants were compensated £20 in vouchers for their time. CG (female, Research Fellow, PhD) conducted the interviews and had training as part of a MSc in Research Methods in Psychology. CG was part of the team that developed *Drink Less* which was a potential source of bias.

Interviews were semi-structured and follow-up questions were allowed. The interview guide was pilot tested with members of the research group (who read through and provided feedback on the guide) with no resulting changes. Participants were set four tasks (e.g. set a goal) before being asked to browse the app (see *Extended data* ( https://osf.io/wzvt8/)). No repeat interviews were carried out. Interviews were audio recorded, transcribed verbatim and analysed (by CG) using content analysis, thus enabling us to quantify the data in terms of the frequency of different themes [19]. No field notes were made and transcripts were not returned to participants for comment or correction. The initial set of themes were selected deductively, based on those used previously to classify users’ reviews for software maintenance and evolution (see section on ‘content analysis of user feedback’) [21]. Sub-themes within these categories were then generated inductively from the data. NVivo version 11 ( Taguette is a freely available alternative) and Microsoft Word were used to manage the data. Transcripts were coded against these initial sub-themes in an iterative process that led to new sub-themes being identified or existing sub-themes being amended to accurately capture the essence of the data.

This study found that the ‘information giving’ theme had five sub-themes: positive feedback on the app; easy to use; difficulty with navigation; reactions to normative feedback, and concerns about the game. The ‘information seeking’ theme had six sub-themes: how do I do this; how to I find this; what are the drinking guidelines; why should I trust this data; what is this trying to tell me, and what’s in a drink. There were also nine major feature requests and four problems discovered by participants that all required changes to be made to the app. Participants did not provide feedback on the findings. Participant quotations to illustrate the themes are provided in *Extended data* ( https://osf.io/wzvt8/). Participants generally had positive feedback on the app and found it easy to use, though a number of issues were identified in terms of novice use of the app resulting in changes to *Drink Less* to ensure the refined version of the app has good usability among users from a range of socioeconomic backgrounds.

*Testing for bugs*

Testing for bugs was an extensive and iterative process. Screens that required user input were tested with dummy data and the app was thoroughly examined for programming bugs before being released. Informal testing was performed by members of the research team. Testing included the app’s text, design and functionality, the registration process, and the fidelity of data storage.

## Results

**Figure 2.**
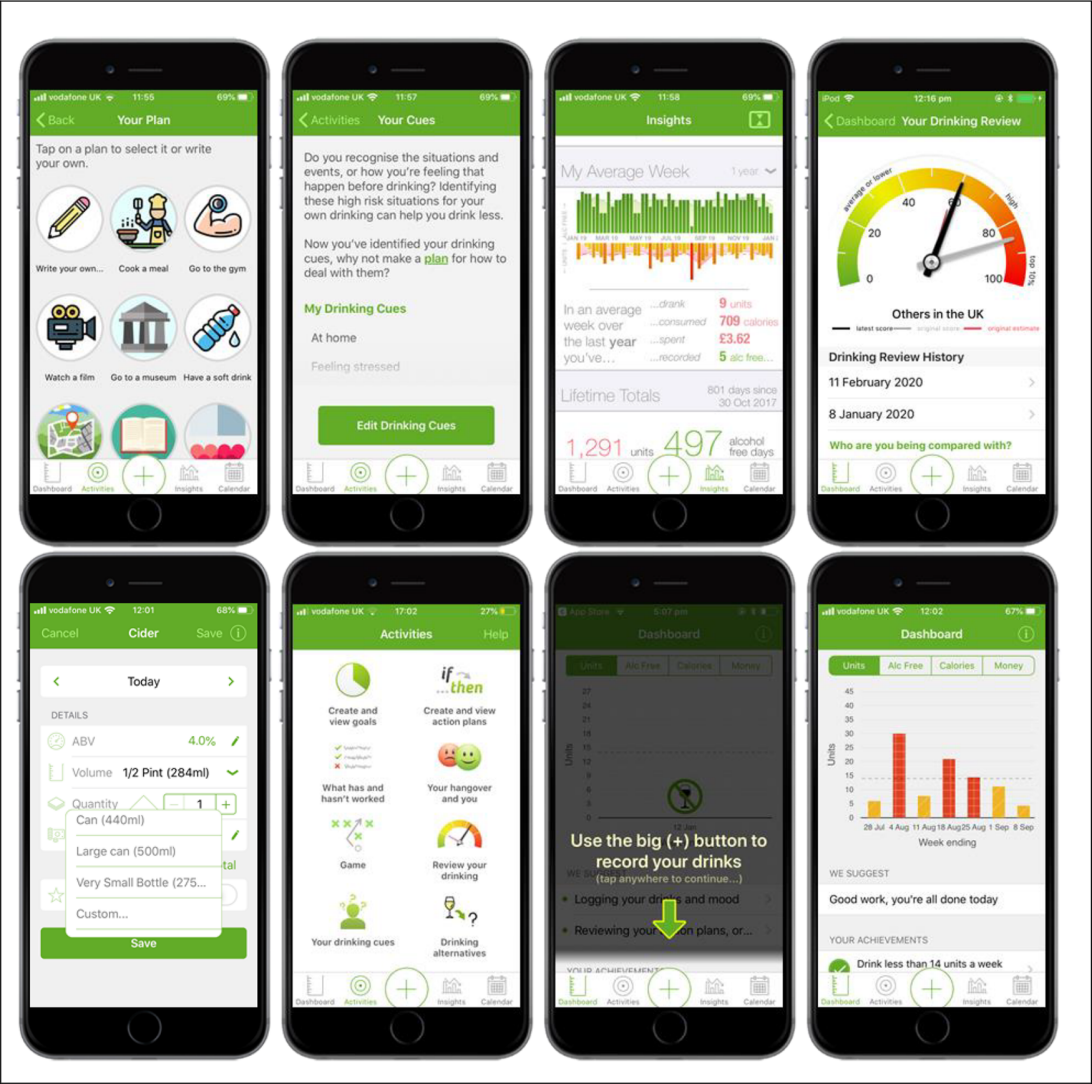
Screenshots from the refined version of the *Drink Less* app.

A number of key changes were made to the content and design of the *Drink Less* app to improve its likely effectiveness and usability, including adding new modules, removing existing ones and making changes to improve existing modules. Example screenshots from the *Drink Less* app of some of these changes are shown in Figure 2.

Table 1 reports how the three phases of the refinement process relate to changes to the app modules. The full content of the refined version of *Drink Less* is available in *Extended data* ( https://osf.io/akqy9/). Supplementary Table 1 provides a summary of the modules and the relevant BCTs for each module, along with whether the modules were present in the original and/or revised version of the app (see *Extended data*: https://osf.io/c3s6j/).

**Table 1.** How the three phases of the refinement process relate to changes to app modules.

App module	Phase 1			Phase 2		Phase 3
	Factorial screening trial	Update of evidence review	User feedback	Public involvement through an Alcohol Discussion Group	Consultation with app designers, developers and researchers	User testing
Behavioural Substitution		Add (original Cochrane Review).		The plans added to be linked to their normal phone calendar. *	Need to reduce amount of text on screen with basic minimal layout.	Positive feedback. Clearer instructions on how to add an alternative plan and why to make one. Longer list of possible options to choose between. Be able to pick multiple options per day. *
Information about Antecedents		Add (updated review).		Did not like the name ‘Drinking triggers’ and would prefer ‘cues’.	Reduce amount of text on screen and simplify.	Positive feedback. Further details explaining the purpose of the module. How it related to other modules – link it to action planning.
Insights			Wanted running totals for alcohol units consumed.	Want a more detailed summary of feedback weekly and/or monthly.	Drop-down menu for different time periods for ‘My Average Week’.	Positive feedback. Preferred the average week graph as a bar graph and for lifetime totals section to be represented in ‘numbers and words’.
Identity Change	Remove.					
Normative Feedback	Retain.		Wanted the ability to update their data used in the normative feedback.	Wanted more information on the data used for the normative feedback and the credibility of this information.	User to be able to update every 28 days. Have a home page with a gauge showing their original and latest scores. List of individual dates below this linking to full normative feedback for that score.	Positive feedback. Surprised by the normative feedback and questioned its credibility. Wanted more information in terms of what the normative feedback and AUDIT feedback meant as they found the wording confusing.
Self-Monitoring and Feedback	Retain.		Wanted calendar week to start on Monday. Not clear how to navigate to drinking calendar. Not clear how to navigate to mood diary. Unsure how to edit or delete drinks entries. Wanted to be able to add customisable volumes. Unclear that you can edit the default settings when adding a new drink. Bug fixes for drinks entry when time zone changed and when clocks went back.		Add an ‘Edit’ button in the top menu bar that when tapped mirrors the ‘delete’ widget in iOS; and add two large save/delete buttons at the bottom of the drinks screen. Start with default setting with the custom option as the last on the drop-down list with user typing in new volume. Any custom volumes added then added to the drop-down list.	Positive feedback. Unsure of how to add a mood diary for the first time. Did not realise that you could edit the type, volume or alcohol by volume (ABV) and that there were drop-down lists. Wanted to know how many calories in a typical drink.
Goal Setting						Positive feedback. How to set a new goal and how to edit existing ones. Did not realise that they could change the type of goal; wanted it to be clearer that there was a drop-down list. Wanted a direct link from goal setting page to drinking guidelines as they were not clear why the default was ‘14’ for the units goal.
Cognitive Bias Re-training	Retain.		Wanted any sugary or fizzy drink images removed from the game.	Wanted the images of sugary drinks to be removed from the game.		Positive feedback. Instructions to be briefer with less text on the first screen.
Action Planning	Retain.					Positive feedback. Your action plans shown on the dashboard. *
Navigational Structure						Difficulty with navigation across the app. Unclear what the difference between the progress and dashboard screens was.
Dashboard						Wanted drinking guidelines easier to find. Suggested list of tasks didn’t make sense as Drink + Me no longer an option.
Onboarding						Needed guidance during onboarding; found the three-step process confusing. Make the link to the infographic on alcohol units more prominent as did not notice it initially.
General			Android OS version of the app. * International availability. * Making the app accessible for individuals with red-green colour blindness. General Data Protection Regulation 2018 changes.	Wanted even less text on the app generally. Tailored reminders. *		Positive feedback. Not clear what the drinking guidelines are or what a unit is.

* Changes not implemented due to budget restrictions.

### Adding ‘Behaviour Substitution’ module

The ‘Behaviour Substitution’ module was added as it was found to be associated with the effectiveness of digital alcohol interventions in the Cochrane Review ( Garnett *et al*., 2018b) (Phase 1). The module design was based on existing interventions and how other apps had implemented a similar concept (Phase 2). The module received positive feedback in user testing, though users wanted clearer instructions on how to add an alternative plan and why to make one, and a longer list of options to choose from (Phase 3). The final ‘Behaviour Substitution’ module, labelled ‘Drinking Alternatives’ in the app, prompts users to make an alternative plan within the drinking calendar on the app (e.g., go for a walk, watch a film, have a soft drink). Users can choose from a list of options or write their own, and set a reminder if they wish to. See Figure 2 and Supplementary Figure 1 (see *Extended data* ( https://osf.io/5y7pc */* ) for screenshots).

### Adding ‘Information about Antecedents’ module

The ‘Information about Antecedents’ module was added as it was found to be associated with effectiveness of digital alcohol interventions in the update of the evidence review (Phase 1). The module design was based on existing interventions and the implementation of this concept in other apps (Phase 2). The final design was chosen based on development costs and simplicity for the user. Public input through an Alcohol Discussion Group suggested the wording ‘cues’ rather than ‘triggers’ (Phase 2). Users requested further details explaining the module’s purpose and how it related to other modules (Phase 3). The final ‘Information about Antecedents’ module, labelled ‘Your Drinking Cues’ in the app, prompts users to identify the situations and events, or feelings that occur before drinking. Users can select from a list of drinking cues and add their own. Users are then prompted to make a plan for how to deal with these drinking cues and signposts the ‘Action Planning’ module. See Figure 2 and Supplementary Figure 2 (see *Extended data* ( https://osf.io/5y7pc/) for screenshots).

### Adding ‘Insights’ module

A common new feature requested in user feedback was a running total for the units of alcohol consumed (Phase 1). Public input included asking for a more detailed summary of weekly and/or monthly feedback (Phase 2). Designs for this new module were based on how other apps had presented similar modules alongside a consultation with app designers, developers, and researchers (Phase 2). During user testing, users provided positive feedback on the ‘Insights’ module and preferred the design as bar graphs (Phase 3). The final ‘Insights’ module, labelled ‘Insights’ in the app, consists of one screen with three sections: My Last Week, My Average Week, and Lifetime Totals. My Last Week and Lifetime Totals showing the total number of units, money spent, calories consumed, and alcohol-free days as well as a summary of their goals. My Average Week shows their average week in terms of units, calories, cost, and alcohol free days over different user-selected time periods: one month, three months, six months, one year, lifetime, with a graphical representation of their weekly units and alcohol free days. See Figure 2 and Supplementary Figure 3 ( *Extended data*: https://osf.io/5y7pc/) for screenshots of the ‘Insights’ module.

### Removing ‘Identity Change’ module

The Bayes factor analysis of additional trial outcome data (Phase 1) indicated that there was support for the null hypothesis that the ‘Identity Change’ module – helping users foster a change from seeing being a “drinker” as a key part of their identity – had no effect on past week alcohol consumption ( Garnett *et al*., 2019b). These findings, alongside results from analysing user feedback (Phase 1) and usage data indicating users very rarely viewed these screens, guided the decision to remove the module. There were many screens within the module that may have meant it was too complex to use, and it may be that, had the intervention content been implemented differently in the app, it would have been more popular with users.

### Changes to ‘Normative Feedback’ module

The ‘Normative Feedback’ module provides feedback on how their own drinking compares with the norms for alcohol consumption and alerts them to any discrepancy. A high-priority change identified from the content analysis of the user feedback was to include the ability for users to update the normative feedback they received (Phase 1). App designers, developers, and researchers were consulted on the best way to implement this change and to illustrate the various user scores (Phase 2). The final design was a new home screen for the module with a dial widget that showed both their original (in grey) and latest (in black) normative feedback. Users could re-take a three-item survey (the AUDIT-C) every 28 days via a link on the Dashboard named “See how your drinking has changed”. Users then saw normative feedback that was tailored to their latest score as well as being able to see any of their previous feedback by tapping on the relevant date under ‘Drinking Review History’ on the main home screen for the module. See Figure 2 and Supplementary Figure 4 ( *Extended data*: https://osf.io/5y7pc/) for the final designs.

During the user testing, some users wanted further clarification about the meaning of the feedback they received from the AUDIT and the Normative Feedback (Phase 3). Therefore, changes to the text on these screens were made to clarify their meaning (see Supplementary Figure 5, *Extended data*: https://osf.io/5y7pc/).

### Changes to the ‘Self-Monitoring and Feedback’ module

The ‘Self-Monitoring and Feedback’ module prompts users to record their alcohol consumption and provides feedback on their consumption, the consequences of consumption, and progress against goals. The content analysis of user feedback found that a high priority change was for the Drinking Calendar week to start on a Monday, instead of a Sunday, to match up with the weekly goals (Phase 1); this was implemented immediately prior to Phase 2. User feedback also showed that users struggled with how to edit or delete an existing drink entry (Phase 1). The consultation with app designers, developers and researchers in Phase 2 resulted in adding an ‘Edit’ button in the top menu bar that mirrors the ‘delete’ widget in iOS (i.e., shows a little red circle which, if tapped, shows the ‘delete’ button from the right), and adding the option to delete the entry, instead of save, when users were editing it. See Supplementary Figure 6 ( *Extended data*: https://osf.io/5y7pc/) for screenshots.

During the user testing, many users did not realise that they could edit the default settings when adding a drink and that there were drop-down lists (Phase 3). Therefore, an arrow was added to indicate that the user could edit these fields. The content analysis of user feedback identified that users wanted the ability to customise volumes when adding a drink (Phase 1). The final design selected during the consultation of app designers, developers, researchers (Phase 2) still had a default start value and a drop-down list of common options but with ‘Custom’ at the end allowing users to type in the exact volume in ml (see Figure 2 and Supplementary Figure 7, *Extended data*: https://osf.io/5y7pc/, for screenshots).

The user testing found that users were unsure of how to add a mood diary for the first time and wanted more guidance on how to use this part of the app (Phase 3). An overlay was added for the Dashboard indicating how to add a mood diary and further text was added to the ‘Your hangover and you’ screen, which appears before any entries are made, and explains how to add one (see Supplementary Figure 8, *Extended data*: https://osf.io/5y7pc/). Other changes from the user testing included highlighting the links to the ‘units infographic’ in bold and to include the typical calories alongside the units in the infographic (Phase 3).

### Changes to ‘Goal Setting’ module

The ‘Goal Setting’ module allows users to set a weekly goal in terms of units, calories, cost and alcohol-free days. In the user testing, users often did not realise that they could change the type of goal they were setting or choose from a drop-down list; therefore, an arrow was added to indicate that users could choose the type of goal (Phase 3). Users also wanted a link to the drinking guidelines from the ‘Set a goal’ screen as they did not understand why the default was ‘14’ for the units goal. Text explaining this was added (Phase 3). See Supplementary Figure 9 ( *Extended data*: https://osf.io/5y7pc/) for screenshots.

### Changes to the ‘Cognitive Bias Re-training’ module

The ‘Cognitive Bias Re-training’ module aims to change approach biases to alcohol stimuli through an approach-avoidance style game in which users must avoid alcohol-related pictures and approach non-alcohol-related pictures. Two changes were made to this module; the first was as a result of the content analysis of user feedback (Phase 1) and the public input from an Alcohol Discussion Group (Phase 2) in which users did not want the game to be promoting other unhealthy drinks as an alternative to alcohol. Therefore, we removed any non-alcohol-related pictures that involved sugary and/or fizzy drinks from the game. The second change arose from the user testing and involved re-ordering the instructions, so the demonstration screens were shown first and then the text screen was last and made briefer (Phase 3). See Supplementary Figure 10 ( *Extended data*: https://osf.io/5y7pc/) for screenshots of these changes.

### Changes to the Dashboard and the app’s navigational structure

During user testing, users requested a link to the Drinking guidelines screen under ‘Useful Links’ on the Dashboard (the main landing page of the app) and that the ‘We suggest’ list was updated to reflect the new app modules (Phase 3). See Supplementary Figure 11 ( *Extended data*: https://osf.io/5y7pc/) for screenshots. An issue for many users during user testing was the navigational structure as some users were confused how to find different modules and by the names of the modules (Phase 3). Therefore, to improve the navigational structure, the navigation tab bar (at the bottom of the screen) was changed to include the app modules visited more frequently; the ‘Calendar’ replaced the ‘Game’ which was moved to the original ‘Progress’ screen (renamed ‘Activities’ to highlight the difference from the Dashboard).

### Guidance on how to use the app during onboarding

Users needed more guidance on how to use the app during onboarding (first time use of the app and set-up) during the user testing (Phase 3). The original onboarding process on the Dashboard involved three steps (set a goal; add drinks; explore) but there was no clear link between the steps or how to find those steps again, resulting in users getting confused and lost on the app. Therefore, the three-step onboarding process on the Dashboard was removed and integrated into the app in different ways to provide more guidance to users. The ‘set a goal’ step was moved to the end of registration in a step-by-step wizard so that all users could choose to set a goal by the time they reach the Dashboard. Then on arriving at the Dashboard, a number of overlays take the users through how to perform the key functions on the app: add a drink; view your drinking calendar; view the graph on the Dashboard; enter your mood diary. See Figure 2 and Supplementary Figure 12 ( *Extended data*: https://osf.io/5y7pc/) for screenshots of these changes.

### General changes

The content analysis of user feedback highlighted a key change to make in terms of making the app accessible for individuals with red-green colour blindness (Phase 1). The original design of the app meant that individuals with red-green colour blindness could not differentiate between these colours on the calendar or Dashboard graphs. On the recommendation of a user, we gave each of the colours a texture (e.g., red = ‘hash’, orange = ‘diagonal’) so that any user could understand the colour scheme of the app (see Figure 2 and Supplementary Figure 13, *Extended data*: https://osf.io/5y7pc/). Finally, changes were made to the ‘Help’ section of the app to update the information provided to users as this was out of date from the first version of the app (e.g., NHS websites, where to find further support).

## Discussion

### Summary of main findings

*Drink Less* is a standalone app for people in the UK who are seeking support to reduce their alcohol consumption. To the authors’ knowledge, this is the first alcohol reduction app that has been refined, following its original development, using a systematic and iterative process based on evidence and user input. The refinement resulted in a number of major changes to the app including the addition of three modules – ‘Behaviour Substitution’, ‘Information about Antecedents’ and ‘Insights’ – and the removal of the ‘Identity Change’ module. ‘Behaviour Substitution’ and ‘Information about Antecedents’ are both behaviour change techniques that were identified from the review of digital alcohol interventions, and users provided feedback on how they were implemented during the user testing. The ‘Identity Change’ module was removed from the app based on the findings from the factorial screening trial which showed there was support for no effect of the module ( Crane *et al*., 2018; Garnett *et al*., 2019b). These findings, alongside user feedback and usage data on the most frequently visited screens, guided the decision to remove the Identity Change module. In response to user feedback and user testing a number of changes were conducted within existing modules to improve the app’s usability and acceptability, and improvements were made to the navigational structure and onboarding process. The refinement of the *Drink Less* app continues to follow MOST for developing and evaluating complex behavioural interventions, and the refined version of the app is now ready for a confirmatory evaluation in an RCT ( Garnett *et al*., 2021b).

### Strengths and limitations

*Drink Less* is the first alcohol reduction app aimed at the general population of people who are hazardous or harmful drinkers to have reported a systematic and transparent approach to its development, screening and refinement. Transparency with both the process and content of the intervention is important for the replication of interventions ( de Bruin *et al*., 2021). This paper follows on from the original development paper of *Drink Less* ( Garnett *et al*., 2019a) by reporting the processes and resulting content from refining the app in the next stage in MOST. By refining the app and releasing an updated version, the app has avoided issues of poor user experience and early app abandonment, and not been left to ‘rot’ in the app store, as is the case for many apps developed by academics ( Larsen *et al*., 2016).

One of the strengths of this refinement process was that it drew on empirical evidence and user input to improve the usability and likely effectiveness of the app. The user input – both from novice users (during the user testing) and experienced users (through the user feedback received) – was critical to improve the app’s usability, which is important for longer-term user engagement. Therefore, it is likely that the refined version of *Drink Less* has been further improved in terms of likely effectiveness at reducing alcohol consumption and that users will find it usable and acceptable. This places the *Drink Less* app in a strong position to be evaluated in an RCT, the next step in MOST, which will advance the limited evidence base in this field.

Another strength of this study is the mixed methods approach and the triangulation of different sources of evidence to refine *Drink Less.* For example, the user testing with novice users identified navigation issues with the onboarding process, which were not identified in the analysis of existing user feedback. Users who have difficulty with navigation during the onboarding process in a real-world setting may disengage with the app completely and not provide feedback. The user feedback is unlikely to have a social confirmation bias as it came from a real-world setting, though it may be that user reviews are polarised and therefore not necessarily representative of the majority of users.

The refinement of *Drink Less* continues to be in line with the Open Science principles of making materials, data, results and publications freely available ( Munafò, 2016), which is important for efficient scientific progress and to reduce trial wastage. The content of the refined version of the app is described in *Extended data* ( https://osf.io/akqy9/) and this paper may provide a helpful template to other researchers refining health-related digital interventions.

There are some limitations with this process of refining *Drink Less.* First, we could not know what changes would arise from the process in advance of applying for funding and were limited by a fixed budget that we estimated in advance. The fixed budget was a key factor in how many changes could be implemented which meant we had to prioritise the changes and not every improvement identified, such as tailored reminders, could be implemented (see Table 1 for the changes not implemented due to budget restrictions). Changes arising from the randomised factorial screening trial and the update of the evidence review (in Phase 1) – the addition and removal of modules – were all implemented to improve the likely effectiveness of the app. However, the implementation of some modules was adapted and simplified to keep the cost of development within budget. Changes to the app that arose from the user feedback (Phase 1), public input from an Alcohol Discussion Group (Phase 2), and user testing (Phase 3) were prioritised based on importance to the users (judged on how frequently the suggested change occurred) and balanced with the development cost of making that change, as well as consideration of how many other changes could be made. We acknowledge that the decisions made in prioritising changes by the research team was subjective and that another team may have made different decisions based on the same information.

Cost was a major factor in the decisions made when refining the *Drink Less* app and this is often an issue with the development of digital interventions where an interdisciplinary team with varying expertise is required. Our research budget covered researcher salaries and development costs for a professional app developer and designer. Another way of working is to have an in-house developer which can remove limitations on what changes can be made. However, there is still the cost of the developer’s salary to cover and therefore this may only be good value for money when the team is working on multiple digital interventions. It is also worth noting that development costs are likely to change over time and also depend on the intervention itself. Therefore, the costs associated with the changes made in the refinement process of *Drink Less* are likely to differ if replicated with a different intervention.

Another limitation is that there is not a universal end point to follow when refining a digital intervention and one could continue to iterate indefinitely. Whilst the refinement process has finished, we will continue to collect and monitor the user feedback we receive to identify changes that can be made to improve *Drink Less.*

There were also some limitations with the methods used to identify the changes to the content of the app. The study analysing the data from the extended randomised factorial screening trial with Bayes factors was unable to use the intention-to-treat approach because there was an extremely low follow-up rate (13%) ( Garnett *et al*., 2019b). In this case, the intention-to-treat approach would have meant no change in the great majority of the sample. A limitation of the update of the review of digital alcohol interventions is that there was infrequent use of many behaviour change techniques meaning the possible effects of most behaviour change techniques could not be evaluated. The reviews included in the content analysis of user feedback may be polarised with views at one end of the spectrum (extremely positive or extremely negative) being more likely than users who were indifferent about the app. Therefore, these reviews are not necessarily representative of the majority of app users. In terms of improving the usability of the app, the interviewer was part of the team that developed *Drink Less*, which was a potential source of bias.

### Future research

The refined version of *Drink Less* will be evaluated in an RCT funded by the National Institute for Health Research (NIHR127651; registered on ISRCTN on 29 ^th^ April 2020 https://doi.org/10.1186/ISRCTN64052601). The trial will evaluate whether the digital offer of *Drink Less* (compared with the recommendation to view the NHS alcohol advice webpage) reduces alcohol consumption over the previous week at a six-month follow-up. This will be the largest RCT of a digital alcohol intervention and the first of an alcohol reduction app for the general population in the UK. There is currently little evidence regarding the effectiveness of apps and this research will start to build a robust evidence base on the effectiveness and cost-effectiveness of health-related apps. *Drink Less* is also collecting user engagement and behavioural data of value in understanding its theoretical mechanisms of action ( Garnett *et al*., 2018a), which can help inform future behaviour change interventions. RCTs are important for evaluation of effectiveness and cost-effectiveness of digital health interventions, though are best undertaken when the intervention is stable and can be implemented with high fidelity ( Murray *et al*., 2016), as is the case here. However, there are many considerations and approaches to the development and evaluation of digital health interventions,depending on the aim or research question ( Murray *et al*., 2016) and there are resources available to help people choose the most appropriate evaluation approach ( Public Health England, 2021). Whilst a lot of the core features, navigational structure and module content remain in the refined version, a number of major changes were made to the original version of the app meaning that the overall content of the original and refined versions of the app are not entirely comparable. A previous study has investigated engagement with the original version of the app ( Bell *et al*., 2020) though no research has yet been conducted on engagement with the refined version of the app. It is an empirical question as to whether engagement with the app has changed as a result of the refined version of the app being released and a question to consider for future research.

## Conclusions

The alcohol reduction app *Drink Less* has been systematically developed and refined based on evidence and theory. A mixed methods approach was used to refine *Drink Less*, following its original development ( Garnett *et al*., 2019a), which provided insights into how to improve the likely effectiveness of the intervention whilst also providing users with what they want from the intervention, which is critical for longer-term engagement. This meant the app was not left to ‘rot’ in the app store, as is the case for many apps developed by academics ( Larsen *et al*., 2016) and issues of poor user engagement were avoided. The three-phase refinement process involved adding three new modules to *Drink Less* (‘Behaviour Substitution’, ‘Information about Antecedents’ and ‘Insights’) as well as a number of changes to existing modules to improve its likely effectiveness, and its acceptability and usability to users. *Drink Less* is now ready for evaluation in an RCT to assess its effectiveness and cost-effectiveness at reducing hazardous and harmful alcohol consumption. *Drink Less* is a freely available intervention to people wanting support to reduce their drinking, which, if found to be effective, could be widely recommended resulting in important implications for public health.

## Data availability

### Underlying data

Open Science Framework: Drink Less/Refinement of Drink Less app. https://osf.io/8tqv6 ( Garnett *et al*., 2021a).

This project contains the following underlying data:

- UKCTAS Alcohol Discussion Group - summary notes.docx (summary notes from the PPI Alcohol Discussion Group, https://osf.io/9ca37/).
- Consultation survey data – Round 1.xlsx ( https://osf.io/jxvns/).
- Consultation survey data – Round 2.xlsx ( https://osf.io/vt3ub/).
- Drink Less_extended trial outcomes dataset.sav (additional trial outcome data from the randomised factorial screening trial, https://osf.io/nad4e)

The user feedback data used for content analysis (Phase 1) cannot be made available publicly as consent was not gained from users to share the user feedback data. Interested parties should contact the corresponding author, Claire Garnett ( c.garnett@ucl.ac.uk), for a restricted route of access to the user feedback data after signing a data access agreement confirming the data will be held securely and that no further research will be conducted using this data.

Audio recordings for the PPI Alcohol Discussion Group (Phase 2) cannot be provided as consent was not gained from participants for this purpose. The audio recordings were taken for the sole purpose of informing the summary notes written (provided in *Underlying data*).

Audio recordings and transcripts for the user testing interviews (Phase 3) cannot be made available publicly as consent was not gained from participants for this purpose. Participants did provide consent for their anonymised data to be used by others for future research and interested parties should contact the corresponding author, Claire Garnett ( c.garnett@ucl.ac.uk), to apply for access to the transcripts for further research after signing a data request form.

### Extended data

Open Science Framework: Drink Less/Refinement of Drink Less app. https://doi.org/10.17605/OSF.IO/8TQV6 ( Garnett *et al*., 2021a).

This project contains the following extended data:

- Report on rapid update of Cochrane systematic review on digital alcohol reduction interventions.docx ( https://osf.io/azpc2/)
- User feedback content analysis - report - Nov 2018.docx ( https://osf.io/vdmsk/)
- App wireframes_handout to UKCTAS Alcohol Discussion Group.pdf ( https://osf.io/mu7sw/)
- Round 1 consultation survey.pdf ( https://osf.io/w3tx8/)
- Round 2 consultation survey.pdf ( https://osf.io/4xa73/)
- User testing brief report_OSF.docx ( https://osf.io/wzvt8/)
- Content of Drink Less app (version 2.0.7).pdf ( https://osf.io/akqy9/)
- Supplementary Table 1.pdf ( https://osf.io/c3s6j/)
- Supplementary Figures.pdf ( https://osf.io/5y7pc/)

Data are available under the terms of the Creative Commons Attribution 4.0 International license (CC-BY 4.0).

## Software availability

- Software available from: https://apps.apple.com/gb/app/drink-less/id1020579244
- Source code available from: https://github.com/UCL-Centre-for-Behaviour-Change/DrinkLess-public
- Archived source code at time of publication: http://doi.org/10.5281/zenodo.4721332 ( Pointryogi & UCL-Centre-for-Behaviour-Change, 2021).

License: GNU Affero General Public License version 3 (AGPL-3.0)
