# Supplementary material for: Refining the content and design of an alcohol reduction app, Drink Less, to improve its usability and effectiveness: a mixed methods approach
Source: F1000Res. 2021 Nov 25;10:511. Originally published 2021 Jun 29. [Version 2] doi: 10.12688/f1000research.51416.2 (PMC8431211; doi:10.12688/f1000research.51416.2)
Supplement: Peer review history

# Peer review: Reviewer response for version 1

**DOI:** 10.5256/f1000research.54583.r92034

**Published:** 2021-09-09

**Authors:** Maria Lucia O. Souza Formigoni

**Affiliations:** 1 Department of Psychobiology, Paulista School of Medicine, Federal University of São Paulo, UNIFESP, São Paulo, Brazil

Although there are several applications (app) to reduce the consumption of cigarettes or alcohol, many were developed based on clinical principles, not always supported by a solid theoretical basis. This is not the case with this manuscript, in which the authors clearly support the choice of tools included in the application and describe in a very detailed way all the steps of refinement of the application carried out. The language is clear and precise and several details of the procedures are made available to the reader in the form of supplementary material, which makes it an excellent source of reference for the area. Despite this, the article could benefit from the inclusion of some details such as:

1. What was the criterion used to determine the sample size (n=18) of the 3rd phase of the study? Was it pre-determined or  the authors use another criterion such as “theoretical saturation”?
2. If the authors have data on the app's adherence to the use of the preliminary version and the updated version, it would be appropriate to include and compare them, as well as to include this point in the discussion.
3. The authors could include more explicitly in the discussion the changes they intended to make and that were not possible due to budget limitations, as well as the criteria adopted for inclusion of changes: they were the most accessible, or the most important from the point of view theoretical or the most frequently requested by users? What are the possible impacts of these decisions? This aspect, as well as the fact that one of the authors was the interviewer, which is considered a bias in the methodology, should appear at the end of the manuscript among the limitations of the study.

The discussion could be further developed, both in relation to the theoretical bases that motivated the changes and in relation to the criticisms made by users.  The removal of the “identity change” module and the inclusion of "Information about antecedents", “Behavior substitution” and “Insights” should be more deeply addressed in the discussion, considering the theoretical aspects involved.

Is the work clearly and accurately presented and does it cite the current literature?

Yes

If applicable, is the statistical analysis and its interpretation appropriate?

Yes

Are all the source data underlying the results available to ensure full reproducibility?

Yes

Is the study design appropriate and is the work technically sound?

Yes

Are the conclusions drawn adequately supported by the results?

Yes

Are sufficient details of methods and analysis provided to allow replication by others?

Yes

Reviewer Expertise:

brief interventions and virtual interventions

I confirm that I have read this submission and believe that I have an appropriate level of expertise to confirm that it is of an acceptable scientific standard.

# Response

**Published:** 2021-11-22

**Authors:** Claire Garnett

**Affiliations:** University College London, UK

- *Although there are several applications (app) to reduce the consumption of cigarettes or alcohol, many were developed based on clinical principles, not always supported by a solid theoretical basis. This is not the case with this manuscript, in which the authors clearly support the choice of tools included in the application and describe in a very detailed way all the steps of refinement of the application carried out. The language is clear and precise and several details of the procedures are made available to the reader in the form of supplementary material, which makes it an excellent source of reference for the area.*
- **Response: **Thank you for your positive review and helpful comments which we’ve addressed below.
- *Despite this, the article could benefit from the inclusion of some details such.* *What was the criterion used to determine the sample size (n=18) of the 3rd phase of the study? Was it pre-determined or the authors use another criterion such as “theoretical saturation”?*
- **Response: **A sample size of 16 participants is recommended for a usability test to detect most of the usability problems (Nielsen, 1994). We have clarified about the sample size on page 14: “There were 18 participants that took part, which met the threshold recommended for a usability test to detect most of the usability problems (Nielsen, 1994).”
- *If the authors have data on the app's adherence to the use of the preliminary version and the updated version, it would be appropriate to include and compare them, as well as to include this point in the discussion.*
- **Response:** A previous study investigated engagement with the original version of the app and we have now included this in the discussion on page 27 along with highlighting that future research should consider comparing engagement between the original and refined version of the app: “A number of major changes were made to the original version of the app meaning that the overall content of the original and refined versions of the app are not directly comparable. A previous study has investigated engagement with the original version of the app (Bell et al., 2020) though no research has yet been conducted on engagement with the refined version of the app. It is an empirical question as to whether engagement with the app has changed as a result of the refined version of the app being released and a question to consider for future research.”
- *The authors could include more explicitly in the discussion the changes they intended to make and that were not possible due to budget limitations, as well as the criteria adopted for inclusion of changes: they were the most accessible, or the most important from the point of view theoretical or the most frequently requested by users? What are the possible impacts of these decisions? This aspect, as well as the fact that one of the authors was the interviewer, which is considered a bias in the methodology, should appear at the end of the manuscript among the limitations of the study.*
- **Response: **Thank you, we have added a more detailed discussion of the limitations including clarifying about which changes were not possible due to budget restrictions on p27 and p28: “The fixed budget was a key factor in how many changes could be implemented, which meant we had to prioritise the changes and not every improvement identified, such as tailored reminders, could be implemented (see Table 1 for the changes not implemented due to budget restrictions). Changes arising from the randomised factorial screening trial and the update of the evidence review (in Phase 1) – the addition and removal of modules – were all implemented to improve the likely effectiveness of the app. However, the implementation of some modules was adapted and simplified to keep the cost of development within budget. Changes to the app that arose from the user feedback (Phase 1), public input from an Alcohol Discussion Group (Phase 2), and user testing (Phase 3) were prioritised based on importance to the users (judged on how frequently the suggested change occurred) and balanced with the development cost of making that change, as well as consideration of how many other changes could be made.” “There were also some limitations with the methods used to identify the changes to the content of the app. The study analysing the data from the extended randomised factorial screening trial with Bayes factors was unable to use the intention-to-treat approach because there was an extremely low follow-up rate (13%) (Garnett et al., 2019b). In this case, the intention-to-treat approach would have meant no change in the great majority of the sample. A limitation of the update of the review of digital alcohol interventions is that there was infrequent use of many behaviour change techniques meaning the possible effects of most behaviour change techniques could not be evaluated. The reviews included in the content analysis of user feedback may be polarised with views at one end of the spectrum (extremely positive or extremely negative) being more likely than users who were indifferent about the app. Therefore, these reviews are not necessarily representative of the majority of app users. In terms of improving the usability of the app, the interviewer was part of the team that developed Drink Less, which was a potential source of bias.”
- *The discussion could be further developed, both in relation to the theoretical bases that motivated the changes and in relation to the criticisms made by users.  The removal of the “identity change” module and the inclusion of "Information about antecedents", “Behavior substitution” and “Insights” should be more deeply addressed in the discussion, considering the theoretical aspects involved.*
- **Response:** We have developed our discussion further around these areas, in particular on p25: “The refinement resulted in a number of major changes to the app including the addition of three modules – ‘Behaviour Substitution’, ‘Information about Antecedents’ and ‘Insights’ – and the removal of the ‘Identity Change’ module. ‘Behaviour Substitution’ and ‘Information about Antecedents’ are both behaviour change techniques that were identified from the review of digital alcohol interventions, and users provided feedback on how they were implemented during the user testing. The ‘Identity Change’ module was removed from the app based on the findings from the factorial screening trial which showed there was support for no effect of the module (Crane et al., 2018; Garnett et al., 2019b). These findings, alongside user feedback and usage data on the most frequently visited screens, guided the decision to remove the Identity Change module.”

# Peer review: Reviewer response for version 1

**DOI:** 10.5256/f1000research.54583.r88560

**Published:** 2021-07-15

**Authors:** Andre Bedendo

**Affiliations:** 1 Department of Health Sciences, University of York, York, UK

The paper describes the process of refining the app *Drink Less, *designed to help hazardous or harmful UK drinkers to reduce alcohol consumption. The manuscript is well-written and provides a detailed description of the updating process of the app, an aspect that is often vaguely described or absent in scientific literature of drug digital interventions. This is the main contribution of the study to the field and it is highly appreciated the efforts of the authors in documenting and summarising the app revising/updating process.

 The authors used a three-phase process to refine the app: identified changes to be made, updated the app, and tested the refined version. A mixed-methods approach was utilised and included a factorial randomised trial, content analysis of user feedback, expert consultation, public involvement group, Cochrane systematic review, semi-structured interviews, etc. The manuscript also presents multiple information as supporting files (e.g. data, user’s feedbacks, app prototypes, etc.). The study did not present a typical discussion section with results being discussed in light of other research, but the authors commented on various strengths and limitations. Finally, the overall quality of the manuscript is good, and I provide below some minor comments aiming at contributing to the study.

  

1. It is not clear in the manuscript if the results of the randomised factorial trial after the additional data collection are available. Did the additional data change the results observed among the 672 participants? If this information is available, it would be relevant to include it in the manuscript.
2. How the authors defined that the number of 18 participants was sufficient for Phase 3? Also, were participants guided to use all app features or were they free to use during the thinking aloud?
3. Having a table showing the modules that were present in the first version of the app, in the revised version would facilitate reading and provide a good summary to readers. If possible, also include the behaviour change techniques of each module.
4. Considering that major changes occurred in the new iteration of the app, how comparable the two versions of the app are?
5. As highlighted by the authors, engagement is a major issue on digital interventions. Therefore, including details on the engagement of the previous version of the app will be of value as well as to have the authors comment on the potential impact of the inclusion of more modules on the user engagement.
6. The authors highlighted that budget limitations played an important role during the app refinement. However, do authors perceive any limitations regarding the methods used? Similarly, in the future studies section, the authors focused on the presentation of the future RCT evaluation of the app. However, are there any broader recommendations for future studies that also develop and evaluate?

Is the work clearly and accurately presented and does it cite the current literature?

Yes

If applicable, is the statistical analysis and its interpretation appropriate?

Not applicable

Are all the source data underlying the results available to ensure full reproducibility?

Yes

Is the study design appropriate and is the work technically sound?

Yes

Are the conclusions drawn adequately supported by the results?

Yes

Are sufficient details of methods and analysis provided to allow replication by others?

Yes

Reviewer Expertise:

Alcohol use and digital interventions

I confirm that I have read this submission and believe that I have an appropriate level of expertise to confirm that it is of an acceptable scientific standard.

# Response

**Published:** 2021-11-22

**Authors:** Claire Garnett

**Affiliations:** University College London, UK

- *The paper describes the process of refining the app Drink Less, designed to help hazardous or harmful UK drinkers to reduce alcohol consumption. The manuscript is well-written and provides a detailed description of the updating process of the app, an aspect that is often vaguely described or absent in scientific literature of drug digital interventions. This is the main contribution of the study to the field and it is highly appreciated the efforts of the authors in documenting and summarizing the app revising/updating process. The authors used a three-phase process to refine the app: identified changes to be made, updated the app, and tested the refined version. A mixed-methods approach was utilized and included a factorial randomized trial, content analysis of user feedback, expert consultation, public involvement group, Cochrane systematic review, semi-structured interviews, etc. The manuscript also presents multiple information as supporting files (e.g. data, user’s feedbacks, app prototypes, etc.). The study did not present a typical discussion section with results being discussed in light of other research, but the authors commented on various strengths and limitations. Finally, the overall quality of the manuscript is good, and I provide below some minor comments aiming at contributing to the study.*
- **Response: **Thank you for your helpful and thorough comments. We have addressed each of them below.
- *It is not clear in the manuscript if the results of the randomised factorial trial after the additional data collection are available. Did the additional data change the results observed among the 672 participants? If this information is available, it would be relevant to include it in the manuscript.*
- **Response: **We have clarified that on page 8: “In the factorial trial, Bayes factors showed the data to be insensitive to detect a main effect (d=0.22) of individual modules so an update was conducted to analyse additional trial outcome data collected through extended recruitment. This update is published in full elsewhere (Garnett et al., 2019b).”
- *How the authors defined that the number of 18 participants was sufficient for Phase 3? Also, were participants guided to use all app features or were they free to use during the thinking aloud?*
- **Response: **We have clarified the sample size and added details on the think-aloud procedure on pages 14 and 15, respectively: “There were 18 participants that took part, which met the threshold recommended for a usability test to detect most of the usability problems (Nielsen, 1994).” “Participants were set four tasks (e.g. set a goal) before being asked to browse the app (see *Extended data* ( https://osf.io/wzvt8/)).”
- *Having a table showing the modules that were present in the first version of the app, in the revised version would facilitate reading and provide a good summary to readers. If possible, also include the behaviour change techniques of each module.*
- **Response: **This is an excellent suggestion and we have created a supplementary table and referred to this on page 16 of the manuscript: “Supplementary Table 1 provides a summary of the modules and the relevant BCTs for each module, along with whether the modules were present in the original and/or revised version of the app (see *Extended data: *https://osf.io/c3s6j/).”
- *Considering that major changes occurred in the new iteration of the app, how comparable the two versions of the app are?* *As highlighted by the authors, engagement is a major issue on digital interventions. Therefore, including details on the engagement of the previous version of the app will be of value as well as to have the authors comment on the potential impact of the inclusion of more modules on the user engagement. *
- **Response:** These are both good points and we have now addressed them in the discussion on page 30: “Whilst a lot of the core features, navigational structure and module content remain in the refined version, a number of major changes were made to the original version of the app meaning that the overall content of the original and refined versions of the app are not entirely comparable. A previous study has investigated engagement with the original version of the app (Bell et al., 2020) though no research has yet been conducted on engagement with the refined version of the app. It is an empirical question as to whether engagement with the app has changed as a result of the refined version of the app being released and a question to consider for future research.”
- *The authors highlighted that budget limitations played an important role during the app refinement. However, do authors perceive any limitations regarding the methods used? Similarly, in the future studies section, the authors focused on the presentation of the future RCT evaluation of the app. However, are there any broader recommendations for future studies that also develop and evaluate?*
- **Response: **This is an excellent point and we’ve added another paragraph on p28 on the limitations with the individual methods used: “There were also some limitations with the methods used to identify the changes to the content of the app. The study analysing the data from the extended randomised factorial screening trial with Bayes factors was unable to use the intention-to-treat approach because there was an extremely low follow-up rate (13%) (Garnett et al., 2019b). In this case, the intention-to-treat approach would have meant no change in the great majority of the sample. A limitation of the update of the review of digital alcohol interventions is that there was infrequent use of many behaviour change techniques meaning the possible effects of most behaviour change techniques could not be evaluated. The reviews included in the content analysis of user feedback may be polarised with views at one end of the spectrum (extremely positive or extremely negative) being more likely than users who were indifferent about the app. Therefore, these reviews are not necessarily representative of the majority of app users. In terms of improving the usability of the app, the interviewer was part of the team that developed Drink Less, which was a potential source of bias.” It is difficult to provide very broad recommendations for future studies as it will depend on the digital intervention and the aim of the evaluation. We have added a sentence highlighting some resources for deciding on the most appropriate evaluation approach on p29: “RCTs are important for evaluation of effectiveness and cost-effectiveness of digital health interventions, though are best undertaken when the intervention is stable and can be implemented with high fidelity (Murray et al., 2016 ), as is the case here. However, there are many considerations and approaches to the development and evaluation of digital health interventions, depending on the aim or research question (Murray et al., 2016) and there are resources available to help people choose the most appropriate evaluation approach Public Health England, 2021).”
